# Continuous monitoring of chlorophyll *a* fluorescence and microclimatic conditions reveals warming-induced physiological damage in biocrust-forming lichens

**DOI:** 10.1007/s11104-022-05686-w

**Published:** 2022-09-09

**Authors:** José Raggio, David S. Pescador, Beatriz Gozalo, Victoria Ochoa, Enrique Valencia, Leopoldo G. Sancho, Fernando T. Maestre

**Affiliations:** 1 Departamento de Farmacología, Farmacognosia y Botánica, Facultad de Farmacia, Universidad Complutense de Madrid, Madrid, Spain; 2 Departamento de Biología y Geología, Física y Química Inorgánica, Universidad Rey Juan Carlos, Móstoles, Madrid, Spain; 3 Instituto Multidisciplinar para el Estudio del Medio “Ramon Margalef”, Universidad de Alicante, Carretera de San Vicente del Raspeig s/n, 03690 San Vicente del Raspeig, Spain; 4 Departamento de Ecología, Universidad de Alicante, Carretera de San Vicente del Raspeig s/n, 03690 San Vicente del Raspeig, Spain

**Keywords:** Drylands, Global change ecology, Lichen physiology, Photosynthesis, Plant-soil interactions, Soil erosion control

## Abstract

**Purpose:**

Biocrust communities, which are important regulators of multiple ecosystem functions in drylands, are highly sensitive to climate change. There is growing evidence of the negative impacts of warming on the performance of biocrust constituents like lichens in the field. Here, we aim to understand the physiological basis behind this pattern.

**Methods:**

Using a unique manipulative climate change experiment, we monitored every 30 minutes and for 9 months the chlorophyll *a* fluorescence and microclimatic conditions (lichen surface temperature, relative moisture and photosynthetically active radiation) of *Psora decipiens*, a key biocrust constituent in drylands worldwide. This long-term monitoring resulted in 11,847 records at the thallus-level, which allowed us to evaluate the impacts of ~2.3 °C simulated warming treatment on the physiology of *Psora* at an unprecedented level of detail.

**Results:**

Simulated warming and the associated decrease in relative moisture promoted by this treatment negatively impacted the physiology of *Psora*, especially during the diurnal period of the spring, when conditions are warmer and drier. These impacts were driven by a mechanism based on the reduction of the length of the periods allowing net photosynthesis, and by declines in Yield and *Fv/Fm* under simulated warming.

**Conclusion:**

Our study reveals the physiological basis explaining observed negative impacts of ongoing global warming on biocrust-forming lichens in the field. The functional response observed could limit the growth and cover of biocrust-forming lichens in drylands in the long-term, negatively impacting in key soil attributes such as biogeochemical cycles, water balance, biological activity and ability of controlling erosion.

**Supplementary Information:**

The online version contains supplementary material available at 10.1007/s11104-022-05686-w.

## Introduction

Biocrusts are complex soil surface communities formed by autotrophic (lichens, cyanobacteria, green algae and/or mosses) and heterotrophic (fungi, archaea and bacteria) organisms that can be found across terrestrial ecosystems worldwide, but are particularly prevalent in drylands and other resourced-limited ecosystems (Belnap 2006; Castillo-Monroy et al. 2010; Maestre et al. 2011). Biocrusts play key roles in these ecosystems by regulating carbon, nitrogen and water fluxes, by affecting albedo and soil temperature and by serving as habitats of a wide variety of soil biota (protozoa, nematodes, tardigrades, rotifers, mites, collembolans, arthropods and mollusks; Belnap 2006; Castillo-Monroy et al. 2010; Couradeau et al. 2016; Maestre et al. 2011; Rutherford et al. 2017). Significantly, it is well known that all biocrusts components are useful soil stabilizers, especially lichens and bryophytes inhabiting the uppermost soil layers, with their biomass having a close relationship with soil protection ability against erosion (Belnap and Büdel 2016). Drylands, where biocrusts represent a fundamental biotic component covering up to 80% of the surface (Chen et al. 2020), are threatened by ongoing increases in aridity driven by global warming (Huang et al. 2017), which could trigger abrupt ecosystem responses accelerating their degradation and desertification (Berdugo et al. 2020). It is therefore essential to understand how climate change will affect biocrusts and the multiple ecosystem services they provide (López-Rodríguez et al. 2020) in drylands, which currently covering ~41% of the terrestrial surface (Cherlet et al. 2018).

Biocrust constituents like mosses and lichens are poikilohydric organisms very sensitive to changing environmental conditions (López-Rodríguez et al. 2020). In habitats such as drylands, their survival relies on a delicate equilibrium between the tolerance to high temperatures and radiation and the need of occasionally getting wet to prevent severe structural harm due to prolonged desiccation (Green et al. 2011; Wu et al. 2013). Studies conducted over the last decade have shown that warming and altered rainfall regimes have a negative effect on the cover, diversity and photosynthetic capacity of biocrust communities dominated by mosses and lichens (Ferrenberg et al. 2015; Guan et al. 2019; Ladrón de Guevara et al. 2018; Ladrón de Guevara et al. 2014; Maestre et al. 2015; Maestre et al. 2013; Maphangwa et al. 2012). However, there are still important gaps in our understanding of the mechanisms underlying the negative effects of warming on the physiological performance (e.g., reduction of the length of the metabolic activity period and derived effects) and growth of biocrust constituents (Reed et al. 2016). Filling these knowledge gaps is crucial to better forecast how biocrust communities will be affected by ongoing climate change.

Chlorophyll *a* fluorescence is an indicator of physiological performance widely used to monitor biocrust-forming lichens and mosses (Green and Proctor 2016; Lan et al. 2012; Lange 2001; Wu et al. 2013). It provides key information about how these organisms adapt to particular environmental conditions and respond to abiotic stressors like high radiation, drought, and large temperature changes (Gauslaa and Solhaug 2000). When measured continuously, the use of chlorophyll *a* fluorescence allows estimating the amount of time mosses and lichens are metabolically active, a key parameter to understand their carbon balance and to assess stress levels in poikilohydric organisms (Maphangwa et al. 2012). Nevertheless, its relevance under field conditions has been rarely quantified (Lange 2003; Raggio et al. 2017; Schroeter et al. 2010). For example, Raggio et al. (2017) showed that the percentage of metabolic activity of biocrust constituents (understood as the fraction of time that the sample is active on a temporal basis regardless of the intensity of that activity) was strongly related to air relative moisture and temperature across a wide environmental gradient in Europe. However, how ongoing climate change will affect the amount of time that biocrust constituents remain metabolically active (Sancho et al. 2016), and the physiological and growth consequences of these changes, is a relevant and timely question that has not been explored yet.

Here, we combine a unique biocrust manipulative experiment with the continuous monitoring of chlorophyll *a* fluorescence and microclimatic conditions to evaluate how a simulated warming treatment (average temperature increase of ~2.3 °C) affects the metabolic activity and physiological traits linked to photosystem II (PSII) efficiency of the biocrust-forming lichen *Psora decipiens* (Hedw.) Hoffm. This is a cosmopolitan species used in multiple physiological and ecological studies with biocrusts (Colesie et al. 2017; Leavitt et al. 2018; Maestre et al. 2012; Raggio et al. 2018; Ruprecht et al. 2014), so is a good candidate to gain a deeper understanding of the physiological mechanisms behind the impacts of global warming on biocrust-forming lichens. Besides, this biocrust model species is able of colonizing soils with different characteristics and properties (Büdel et al. 2014), providing then potentially useful conclusions over biocrust-soil interactions in a wider range of soil types. There are evidences highlighting that simulated warming treatments do not allow an homogeneous temperature control under different environmental conditions (Reed et al. 2016). Thus, the evaluation of temperature and moisture changes at different temporal resolution (e.g., diurnal/nocturnal and seasonal periods) on the physiological response of biocrust-forming lichens to experimental warming seems to be a relevant question. Our objectives were to: (1) assess how simulated warming affects both the amount of time *Psora* is metabolically active and the efficiency of the PSII (Yield and *Fv/Fm*), which are critical determinants of photosynthetic activity in lichens (Schreiber et al. 1995), (2) explore the relationships between microclimatic conditions and these metabolic activity attributes, and (3) compare how simulated warming affects these relationships across daily and seasonal periods. Given the poikilohydric character of *Psora*, we hypothesized that its metabolic activity would be closely linked with microclimatic conditions at the thallus level (e.g., lichen surface temperature, relative moisture, and photosynthetically active radiation), with a most deleterious effect of warming under high temperature and radiation conditions.

## Material and methods

### Experimental design

**Fig. 1:**
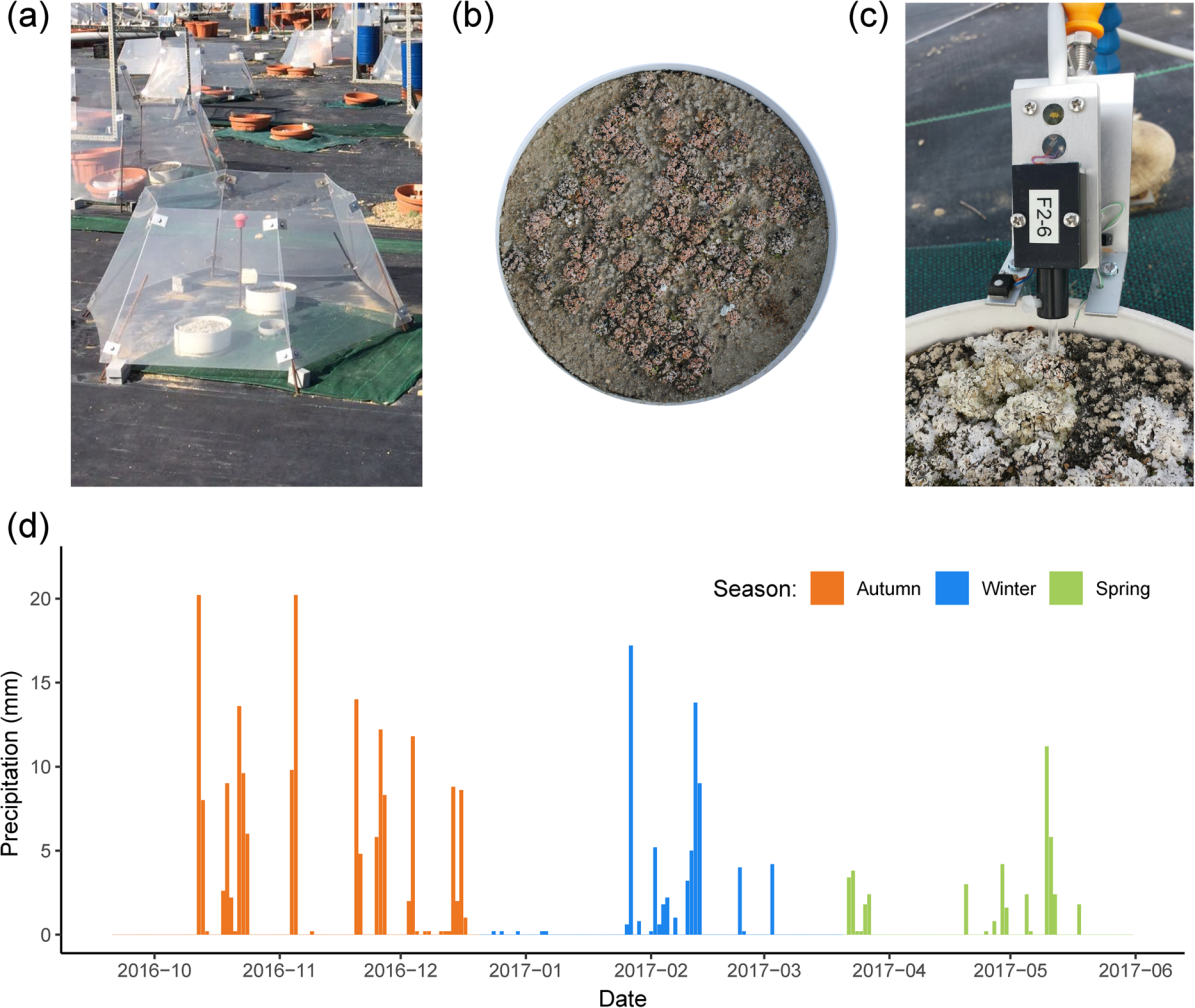
Experiment setup details and pluviogram. **a**) Open top chambers located at the facilities of Rey Juan Carlos University. **b**) A plastic circular pot with squared fragments of *Psora decipiens*. **c**) Close up view of a measuring head of the HexPAM monitoring device. **d**) Daily precipitation (mm) during the experiment

For this study we made use of a unique microcosm experiment conducted at the Climate Change Outdoor Laboratory of Rey Juan Carlos University (CCOL; Móstoles, Spain: 40°20′37′′ N, 3°52′00′′ W, 650 m a.s.l.; Fig. 1a). The climate for this experimental site is Mediterranean-Continental, with mean annual rainfall and temperature values of 365 mm and 15.0 °C, respectively (Getafe, Madrid, Spain: 40°17′58” N, 3°43′20” O, 620 m a.s.l., 1981–2010 30-year period; Spanish Meteorological Agency – AEMET). Diurnal mean temperatures are typically warm in spring (~22 °C), hot in summer (with daytime temperatures rising as high as 40 °C and rarely getting below 30 °C) and cold in winter (~12 °C during the day, ~5 °C at night with lowest temperatures below −4 °C), resulting in fog and harsh frosts during the early-mornings of November, December and January.

The experiment used comprised a fully factorial design with two treatments: lichen richness (three levels: one - monoculture, three and six species mixtures) and warming (two levels: control vs. ~2.3 °C temperature increase). The six lichen species used in the experiment were selected among the most abundant in gypsum environments from the center of Spain (Crespo 1973; Maestre et al. 2005; Martínez et al. 2006): *Buellia zoharyi* (Galun), *Diploschistes diacapsis* (Ach.) Lumbsch, *Fulgensia subbractaceata* (Nyl.) Poelt., *Psora decipiens*, *Squamarina lentigera* (Weber) Poelt. and *Toninia sedifolia*, (Scop.) Timdal. Each monoculture (one species richness level) was replicated 5 times per each warming level, resulting in 10 microcosms per species (60 monoculture microcosms). Three and six species mixtures were replicated 10 times per each warming level, resulting in 40 additional microcosms. For the three species mixtures all possible three-species combinations using the six lichen species were used.

Microcosm consisted of a plastic circular pot (depth 8 cm, internal diameter 20 cm, volume 2.5 l; Fig. 1b) filled with 4.5 cm of homogenized field soil above 3 cm of gravel. Homogeneous 1.1 cm-side square fragments of up to six species were located on the soil surface of each pot to achieve a ~ 60% coverage which is within the range found in the field (Maestre et al. 2005) and following the same random spatial configuration independently of richness level (Fig. 1b).

**Fig. 2:**
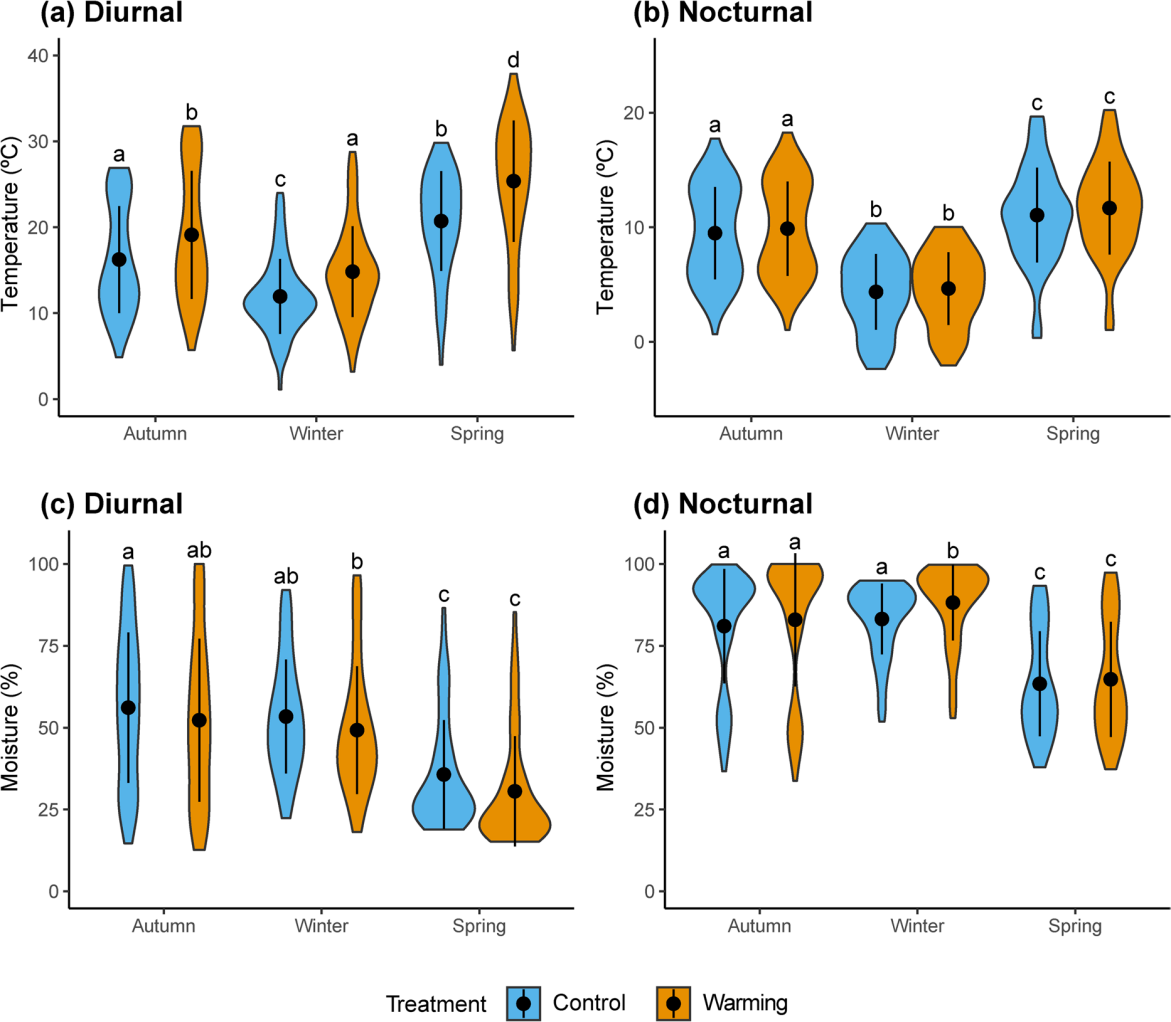
Violin plots showing the distribution of lichen surface temperature (upper panels) and relative moisture (bottom panels) recorded for the diurnal (PAR > 0; left panels) and nocturnal (PAR = 0; right panels) periods across seasons and treatments. Black dots and vertical lines inside each violin represent mean values ± standard deviation of each variable by treatment and season. Letters above each violin plot indicate significant differences (*p* < 0.05, Tukey Contrasts post-hoc test) after a Mixed-Effect model. A total of 1482 observations were used for this analysis

Soil and intact lichen pieces were collected from gypsum outcrops located about 50 km south of the CCOL (lat. 40°02′ N, long. 3°32′ W; 590 m asl; Fig. S1). Lichens were transported to the laboratory on the same day they were collected, cut into square fragments, and sprayed with distilled water twice per week until the setup of the experiment on 1 March 2013 (Fig. 1b). Microcosms were maintained on the soil surface and kept under ambient light, temperature and rainfall, and once lichen fragments were adequately established, we placed them following the fully factorial experiment design with the richness and warming treatment. The warming treatment aims to simulate temperatures predicted for central Spain by the second half of the twenty-first century (i.e., an increase in mean annual temperature ranging between 2.1 and 3.2 °C; Castro et al. 2005; Stocker et al. 2013). To achieve this degree of warming, we used open top chambers (OTCs hereafter), which were built with six methacrylate plates, using a hexagonal design with sloping sides of 65–52-42 cm (Fig. 1a). These chambers have been widely used in multiples studies since the early 90’s for increasing temperatures experimentally and simulating climate warming. Although some studies have discussed some limitations regarding the effect of OTCs over microclimate (e.g., changes over relative moisture, alterations of light conditions or temperature ranges artificially created during the day, especially under warmer conditions and higher radiations; Klein et al. 2005; Aronson and McNulty 2009; Carlyle et al. 2011; Reed et al. 2016) their efficacy to simulate warming has been assessed in diverse studies around the world (e.g., Hollister and Webber 2000, Klanderud and Totland 2005, Escolar et al. 2012 or Elmendorf et al. 2015). Methacrylate used in our OTCs has high transmittance in the visible spectrum (92%), very low emission of the infrared wavelength (4%) and high energy transmission (85%; data provided by the manufacturer, Decorplax Metacrilatos S.L., Madrid, Spain). Also, the OTCs were elevated 5 cm from the soil surface to achieve adequate air flow and avoid excessive overheating. Average temperature increases provided by our experimental OTCs (~2.3 °C), as well as the seasonal variation in such increase (Fig. 2a and b), were within the range predicted by climatic models.

Taking advantage of this experimental design and for the purposes of this study, in September 2016 we selected six thalli of *Psora* located in the edge of six microcosms, being the half of them (three replicates) under ambient (control) conditions and the other half under warming conditions (warming treatment). Owing to the technical limitations (use of an ongoing experiment, availability of monitoring devices due to the high cost of the equipment and length of cables) we had to select relatively close microcosms and with different levels of lichen richness (Table S1). The microcosms selected had almost the same lichen mixtures in both treatments (only 2 of the 6 microcosms are not exactly the same). The low divergence regarding lichen richness between treatments, the fact of having monitored thalli of *Psora* at the edge of the culture (always allowing a free growing way for them not surrounded by other lichens) and the low size of the area being monitored with the fluorescence optic fiber (ca 3 mm) points to a very low relevance of the different lichen richness mentioned in relation with the PSII performance monitored along the study.

### Monitoring of microclimate and metabolic lichen activity

Three microclimatic parameters, the % of time with metabolic activity (% of activity) and the indicators of PSII functioning (Yield and *Fv/Fm*) were monitored using the HexPAM monitoring device (Gademann Instruments, Wuerzburg, Germany), which consists of a central unit that operates the device and records the data of six connected measuring heads (Fig. 1c). Each measuring head has sensors to measure lichen surface temperature (T, °C), relative moisture (%), photosynthetically active radiation (PAR, μmol m^−2^ s^−1^) and metabolic activity using a Pulse-Amplitude-Modulation automatic fluorometer with an optical fiber that is placed from 3 to 5 mm away from each *Psora* thallus (Fig. 1c). Lichen activity was determined with the chlorophyll *a* fluorescence saturation pulse method (see Raggio et al. 2014 and references therein for more details). The effective quantum use efficiency of PSII in the light (Φ_PSII_ or Yield) and its analogue under dark conditions (i.e., *Fv/Fm*) were calculated. To obtain these parameters, each sample was illuminated with a low intensity modulated measuring light, and the resulting chlorophyll *a* fluorescence recorded (*F*_*t*_). Next, each 30 minutes, an actinic flash of high intensity light (4000 μmol m^−2^ s^−1^ during ~1 s) was applied in the same sample point, and the resulting maximal fluorescence recorded (*Fm*, i.e., the fluorescence when PSII is light saturated). Yield was calculated under light conditions (PAR > 0) as $\frac{\left({F}_m-{F}_t\right)}{F_t}$ (Schreiber et al. 1995). This parameter is an indicator of the amount of incident light that goes through the photochemical pathway under conditions of illumination, when the three competitive mechanisms that disperse incident light in a photosynthetic organism (fluorescence, heat dissipation and photochemical pathway) are operative (Maxwell and Johnson 2000). At the same time, *Fv/Fm* (or Yield after pre-adaptation to darkness) was calculated following the same equation used for Yield but under dark conditions (PAR = 0), when all photosystems relaxed. The physiological meaning of *Fv/Fm* is then different, being used as an indicator of potential photosynthetic ability as well as an indicator of the health status of PSII.

To calculate the % of activity on a daily basis, we counted and summed all recorded points with Yield >0 (during the diurnal period) or *Fv/Fm* > 0 (during the nocturnal period) values and calculated a percentage over the total amount of daily data points available (48 recorded points for each day because the machine registered data each 30 minutes). This variable reflects the situations when *Psora* is metabolically active (values = 0 indicate that this species is inactive). At the same time, we calculated a particular daily % of activity considering only light situations above 70 μmol m^−2^ s^−1^. This is a light threshold that ensures the obtention of net photosynthetic values in metabolically active *Psora* samples at a temperature up to 15 °C in a very similar habitat (Raggio et al. 2018). This temperature limit of 15 °C concentrates most of the activity data recorded during the experiment developed here (see results below). Microclimate and metabolic activity were continuously monitored (every 30 minutes) in the six microcosms (three under control conditions vs. three under warming treatment) for 247 days, from 21 September 2016 to 25 May 2017 (when the experiment was harvested), resulting in 11,847 records per *Psora* thallus and microclimatic/physiological parameter. This allows to have a database with a high temporal resolution supplementing that way the replication limitations (*n* = 3 for control conditions and warming treatment) due to the technical constraints described above. Despite the HexPAM monitoring device has an internal filter that directly remove false activity positives based in pre-designed thresholds (10 fluorescence units for Ft and 50 fluorescence unit for Fm are considered as basal noise by default), we removed Yield values <0.1 (from a range of 0 to 0.8 provided by the monitoring device) to ensure that all metabolic activity analysed was clear, robust and not linked to likely drifts in *Ft* and *Fm* due to environmental fluctuations not related with real hydration events of the samples monitored. Similar approaches can be seen at Schroeter et al. (2010) and Colesie et al. (2017).

### Statistical analyses

Repeated measures Linear Mixed-Effect models (LMMs) were applied to test for the main effects of the treatment (two levels: control and warming) and season (three levels: autumn, winter and spring) independent variables on the microclimatic dependent variables (i.e., lichen surface temperature, relative moisture or PAR). For each microclimatic variable, except to the PAR variable, two LMMs were conducted, one for the diurnal period and another for the nocturnal period. Diurnal and nocturnal periods were separated considering the incident PAR, adjusting a threshold of 0 μmol m^−2^ s^−1^ to separate them. Temperature and relative moisture were thus daily averaged for each sensor and period (diurnal/nocturnal). Treatment and season variables were considered fixed factors and microcosm replication was used as a random factor to account for the repeated measures conducted. Pairwise differences of fixed factors (i.e., treatment and season) were identified by multiple comparisons of means (Tukey contrast post-hoc test) on each LMM using an interaction variable including both fixed factors.

The effect of the treatment and season independent variables on the % of activity and mean yield of *Psora* was assessed independently for each of these dependent variables and daily period (diurnal/nocturnal). The % of activity (number of records with positive Yield or *Fv/Fm* values in relation to the total number of records) was obtained for each sensor and daily period. To analyse the % of activity, we fitted two Generalized Linear Mixed-Effect Models (GLMMs) using a binomial distribution, while for the mean Yield, we fitted two LMMs. Microcosm replication was used as a random factor in each model. Pairwise differences of fixed factors were identified by multiple comparisons of means (Tukey contrast post-hoc test) on each Mixed-Effect Model. Additionally, we used the same approach to evaluate the % of activity when PAR conditions were higher than 70 μmol m^−2^ s^−1^.

The effect of microclimatic conditions (i.e., lichen surface temperature and relative moisture) on the % of activity of *Psora* was evaluated independently for each season and daily period (diurnal/nocturnal). This dependent variable (% of activity) and fixed factors (temperature or relative moisture) were averaged as described previously. The interaction of temperature or relative moisture with the treatment variable was also included in the models to assess whether there is an acclimation of *Psora* individuals to the warming treatment. A total of six models (three seasons x two daily periods) per each microclimatic variable (temperature or relative moisture) were fitted. A GLMM based on a binomial distribution was fitted in each case, including the % of activity as dependent variable, the correspondent microclimatic variable and an interaction term including the microclimatic variable and the treatment variable (control vs. warming) as fixed factors, and each replicated microcosm as a random factor.

The packages dplyr (Wickham et al. 2020), lme4 (Bates et al. 2015), multcomp (Hothorn et al. 2008), and ggplot2 (Wickham 2016) were used for data analysis and graph preparation in R version 4.0.5 (R Core Team 2021).

## Results

**Fig. 3:**
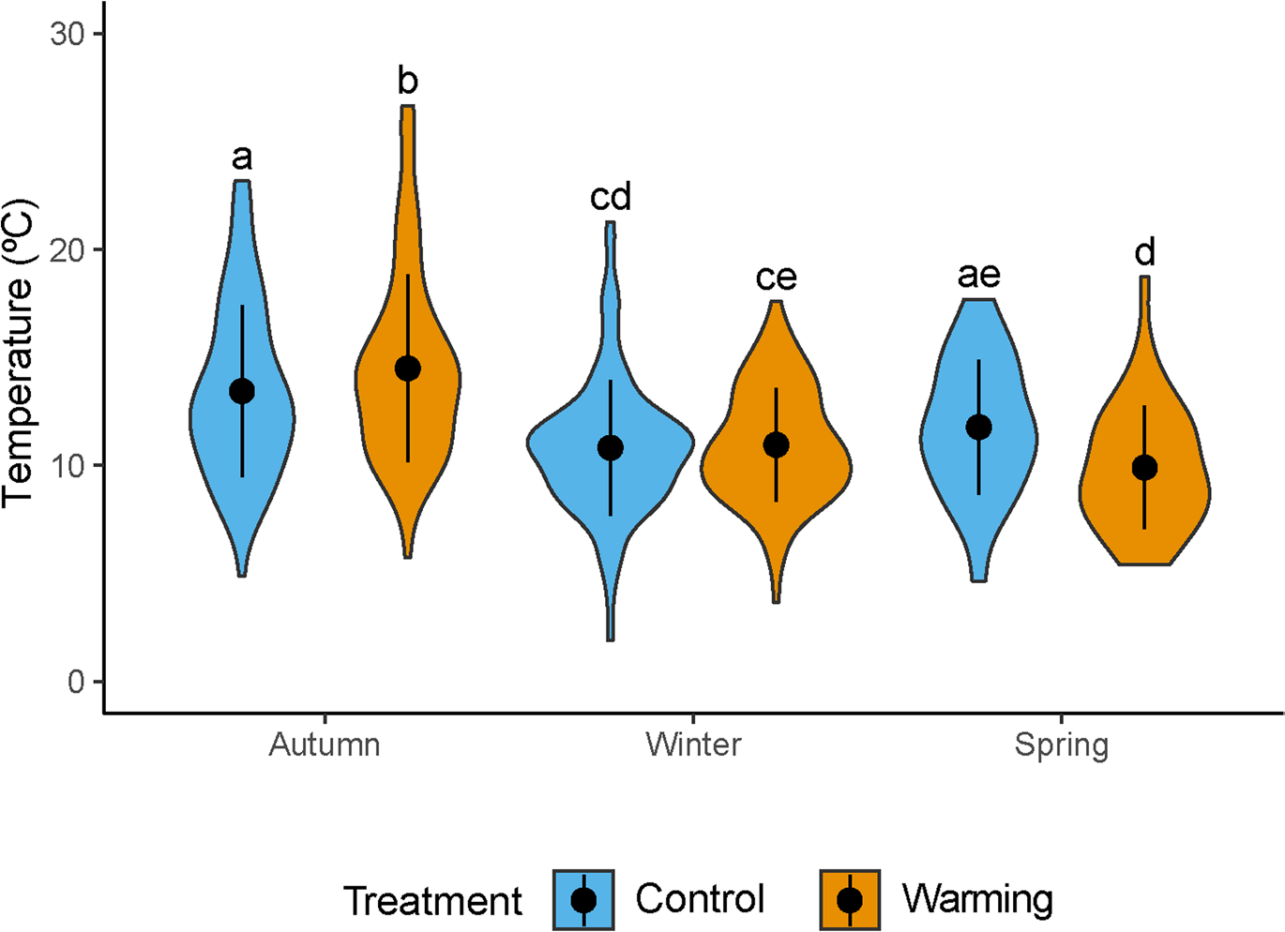
Violin plots showing the distribution of daily lichen surface temperature when the individuals were active (i.e., Yield >0) recorded for the diurnal period (PAR > 0) and for autumn, winter, spring seasons. Black dots and vertical lines inside each violin represent mean lichen surface temperature ± standard deviation by warming treatment level and season. Letters above each violin plot indicate significant differences (*p* < 0.05, Tukey Contrasts post-hoc test) after a Mixed-Effect model. 1111 observations were used for this analysis

The microclimatic parameters recorded at the thallus-level showed differences depending on the treatment, daily period, and season. In general, mean temperature at the lichen surface was significantly higher under warming (diff_Warming-Control_ = 1.65 °C; Fig. 2a, Table S2), particularly during the spring season and under light conditions (Fig. 2a). Importantly, mean temperatures during the night were similar at both control and warming conditions, regardless of the season considered (Fig. 2b). The characteristics of our monitoring allowed us to establish a difference between microclimatic surface temperature during all the monitoring period (Fig. 2a and b) or during activity events only (Fig. 3). When *Psora* was metabolically active, higher mean temperatures were observed in the warming treatment during autumn and winter, but an opposite situation was found during spring (Fig. 3). Mean relative moisture at the thallus level for the whole time series was similar between control and warming conditions (diff_Warming-Control_ = 0.3%; Fig. 2c and d, Table S2). During diurnal periods, however it tended to be higher in the control (diff_Warming-Control_ = −4.75%; Fig. 2c, Table S2), while the opposite pattern was found during nocturnal periods (diff_Warming-Control_ = 3.16%; Fig. 2d, Table S2). However, significant differences between both conditions were only found during the winter nights (Fig. 2d). No significant differences between control and warming conditions were observed in the PAR values recorded for autumn, winter, and spring seasons (Fig. S2).

**Fig. 4:**
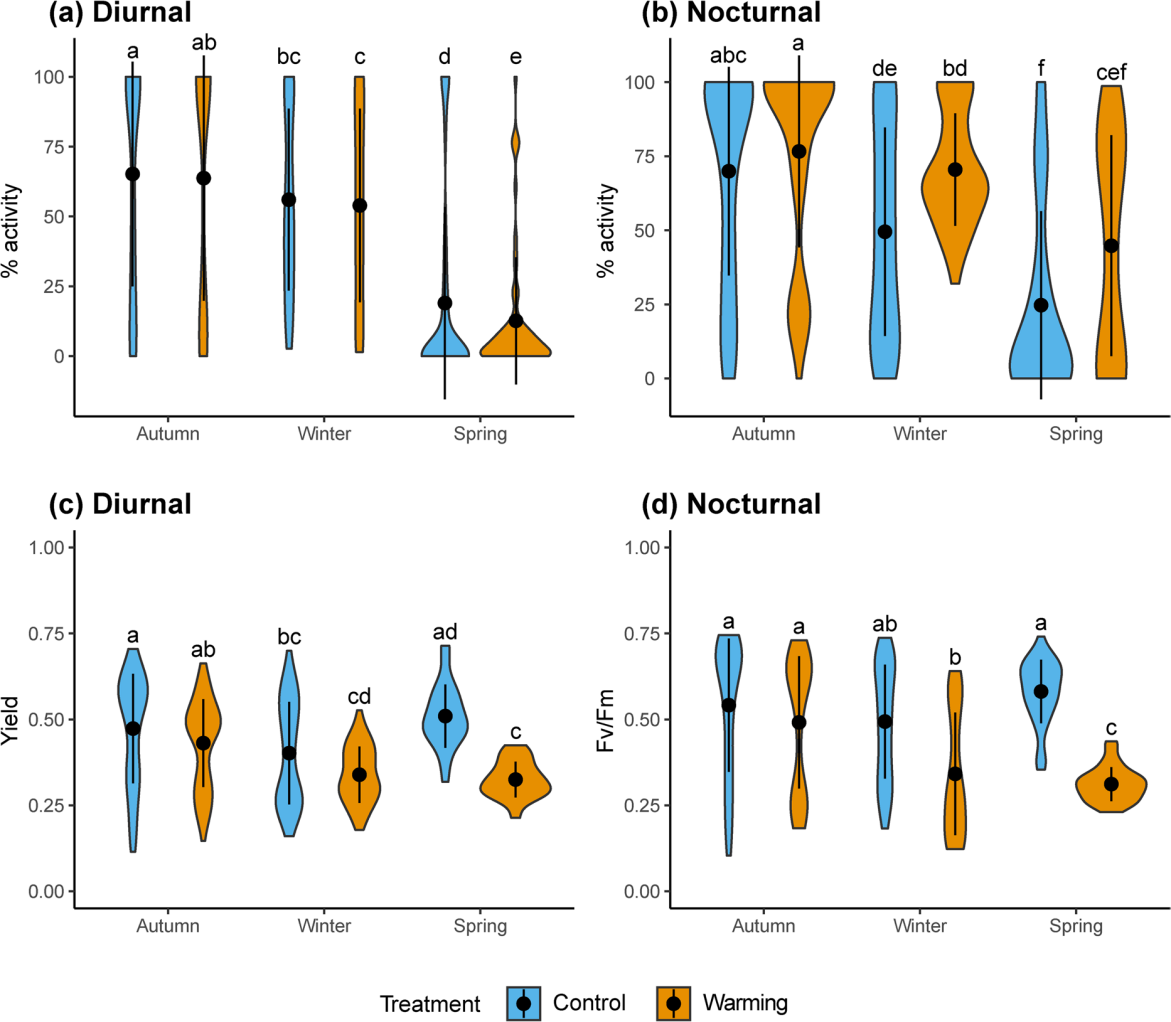
Violin plots showing the distribution of **a** – **b**) daily % of activity, **c**) daily yield and **d**) daily *Fv/Fm* for the diurnal (PAR > 0; left panels) and nocturnal (PAR = 0; right panels) periods and for autumn, winter, spring seasons. Black dots and vertical lines inside each violin represent mean values ± standard deviation of each variable by treatment and season. Letters above each violin plot indicate significant differences (*p* < 0.05, Tukey Contrasts post-hoc test) after a Mixed-Effect model. A total of 1482, 1111 and 1138 observations were used for the analysis of a-b, c and d panels respectively. Daily yield and *Fv/Fm* values result from averaging only positive values (i.e., reflecting the situations where the lichen is metabolically active)

The % of activity of *Psora* under the warming treatment across all seasons was always smaller and higher during the diurnal and nocturnal periods respectively (Fig. 4a and b), but it only shows significant differences between control and warming conditions in spring diurnal period, when the temperature was higher (Fig. 4a). Mean Yield and *Fv/Fm* values showed significant declines under warming from autumn to spring (Fig. 4c and d; Fig. S3). The differences between warming and control conditions were specially marked during the spring both for *Fv/Fm* (when its values were ~ 0.3) and Yield (Fig. 4c and d; Fig. S3). A clear decrease in Yield and *Fv/Fm* was also observed during January (weeks 15–18; Fig. S3) but values raised again in February (week 19; Fig. S3).

**Fig. 5:**
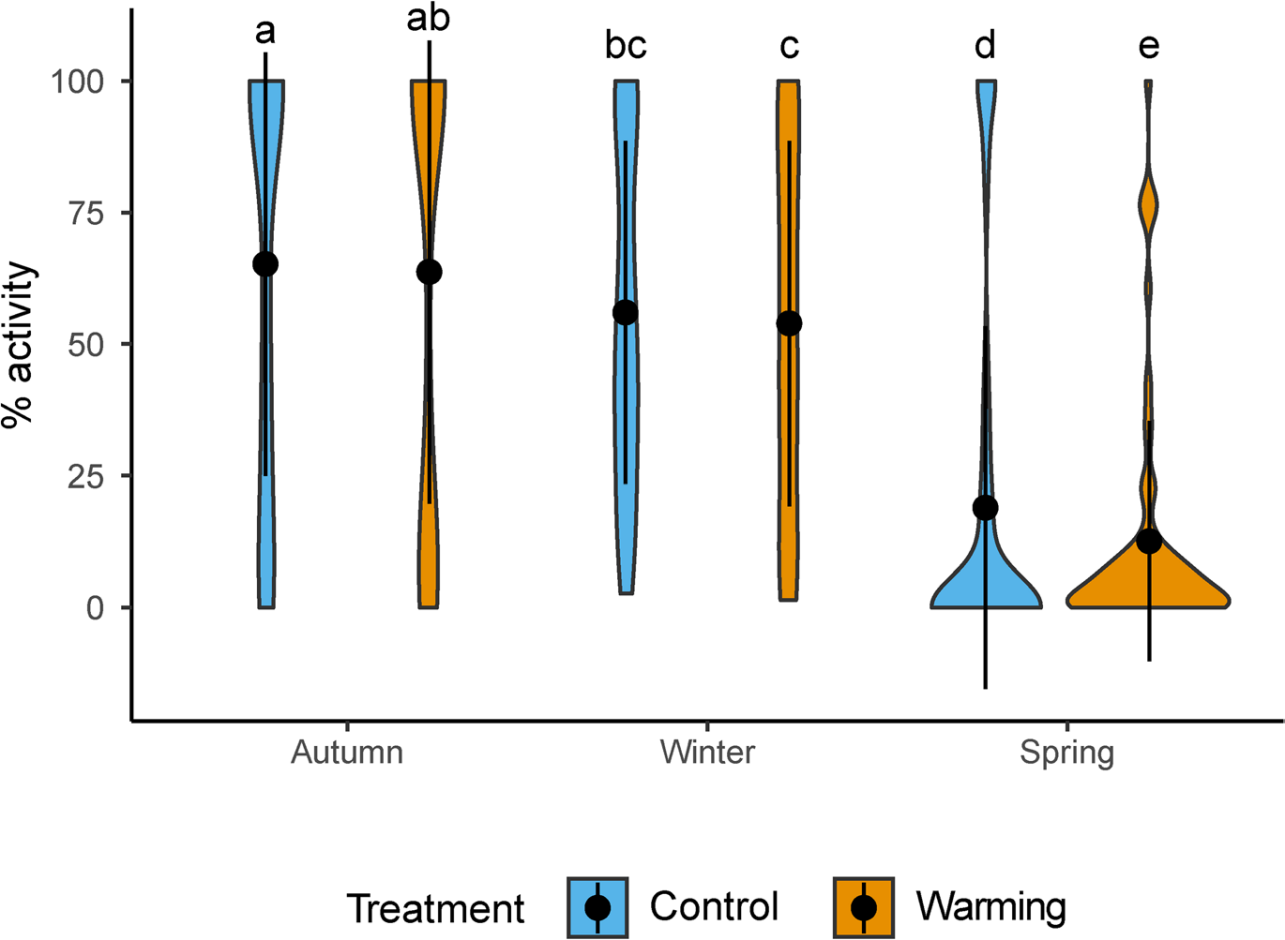
Violin plots showing the distribution of daily % of activity when PAR ≥ 70 for autumn, winter, spring seasons. Black dots and vertical lines inside each violin represent mean daily % of activity ± standard deviation by treatment and season. Letters above each violin plot indicate significant differences (*p* < 0.05, Tukey Contrasts post-hoc test) after a Mixed-Effect model. 1482 observations were used for this analysis)

When mean daily % of activity was analyzed considering PAR conditions higher than 70 μmol m^−2^ s^−1^, we found a higher % of activity in the control conditions throughout the study period, a response that was particularly evident during the spring (Fig. 5).

The proportion of days with a % of activity higher than 50% decreased from autumn to spring (average range from 0.62 to 0.12 respectively; Figs. S4 and S5). The models linking activity with microclimatic variables revealed an increase in the % of activity when temperature decreased (Table 1 and Fig. S4) and moisture increased (Table 1 and Fig. S5). These trends were more evident in the autumn and spring diurnal periods, with intercept values being higher for temperature and smaller for moisture during these periods (Table 1; see Figs. S4 and S5 for a visual interpretation). The slope between temperature and % of activity was more negative under control conditions during these periods. Higher slope values were observed in the warming treatment at night (Table 1 and Fig. S4d and f). It must be noted that when temperatures dropped close or below 0 °C, the % of activity decreased, resulting in very low *Fv/Fm* and Yield values (Figs. S4 and S6). In these cases, we found situations with a positive relationship between temperature and the % of activity (e.g., nocturnal period in winter season; Table 1 and Fig. S4e). The effect of moisture on the % of activity was independent of warming in winter but was smaller under this treatment in spring (Table 1 and Fig. S5c and f).

**Table 1:** Summary of Generalized Linear Mixed-Effects models fitted to link the % of activity with microclimatic variables (i.e., T - lichen surface temperature, and M - relative moisture) for diurnal (PAR > 0) and nocturnal (PAR < 0) periods and for autumn, winter and spring seasons

	Lichen surface temperature (T)			Relative Moisture (M)		
		Diurnal	Nocturnal		Diurnal	Nocturnal
Autumn	Intercept	6.46***	3.96***	Intercept	−4.97***	−6.28***
	T	−0.33***	−0.19***	M	0.12***	0.09***
	T·Treat.	0.04***	−0.14***	M·Treat.	0.001	0.02***
Winter	Intercept	3.47***	0.07	Intercept	−3.14***	−4.13***
	T	−0.20***	0.22***	M	0.07***	0.05***
	T·Treat.	−0.08***	−0.19***	M·Treat.	0.001	0.001
Spring	Intercept	5.19***	3.19***	Intercept	−7.36***	−6.54***
	T	−0.45***	−0.21***	M	0.18***	0.11***
	T·Treat.	0.17***	−0.27***	M·Treat.	−0.09***	−0.04***

Intercept and estimated coefficients of fixed factors used in each model are indicated. Each model includes one of the two microclimatic variables and the interaction term including the correspondent microclimatic and treatment variable (Treat. - control versus warming) as fixed factors and each microcosm replication as a random factor. A binomial distribution was used in all cases. Significant intercepts and fixed factors are marked in bold type and shown as *** *p* < .001; ** *p* < .01; * *p* < .05.

## Discussion

Increases in temperature and reductions in relative moisture promoted by our warming treatment reduced the length of active period in *Psora* during the day, negatively impacting on the status of PSII and suggesting a stress response to this treatment. Our results point to water availability as an important regulator of the effect of warming over the functional performance of *Psora*, and provide a mechanistic physiological explanation for observed declines in the cover of biocrust-forming lichens with warming in field experiments (Ferrenberg et al. 2015; Ladrón de Guevara et al. 2018). Our findings also highlight the value of continuous physiological measurements, and of % of activity in particular, as a tool to monitor the response of biocrust constituents like lichens to ongoing climate change.

Microclimatic conditions experienced by biocrust-forming lichens are key to understand their distribution and adaptation to environmental conditions, especially in habitats with harsh climatic conditions such as drylands (Green et al. 2007). Previous studies have revealed some concerns regarding the effect of simulated warming over microclimate (e.g., Klein et al. 2005; Aronson and McNulty 2009; Reed et al. 2016). The detailed seasonal and diurnal-nocturnal analyses of the microclimatic conditions measured in this study (see Appendix I on the characterization of microclimatic conditions of the warming treatment for more details) indicated that, despite of the unexpected higher moisture found during the winter nights under warming, our experimental treatment provided useful reproductions of likely microenvironmental conditions under natural conditions with ongoing climate change (Bokhorst et al. 2013).

We found a reduction in the % of diurnal activity at the warming treatment across all seasons, which was particularly evident during the spring. This had a clear consequence for the physiology of *Psora*: the reduction of activity events when there were suitable environmental conditions for them. This was particularly supported by the drastic reduction in the % of activity observed above the light threshold ensuring net photosynthesis during the spring (70 μmol m^−2^ s^−1^; Fig. 5). Furthermore, a clear shift in mean diurnal temperatures during activity periods was observed during this season (Fig. 3). While higher diurnal temperatures were recorded during activity conditions under warming for winter and autumn, the opposite pattern was recorded in spring, indicating that the conditions for metabolic activity during the diurnal period were drastically reduced during this season. This pattern confines metabolic activity in spring to cooler situations with lower light availability, thus reducing optimal situations to achieve optimal net photosynthetic values (Fig. 3). Ladrón de Guevara et al. (2018) showed that the limitation of soil surface moisture in artificially warmed biocrusts including *Psora* could underlie the reductions in cover, richness and evenness observed in the field. Colesie et al. (2016) demonstrated that *Psora* could exploit soil water as an extra metabolic activity driver through versatile rhizines. Thus, the warming treatment could have altered the ability to exploit the soil water source in order to keep *Psora* active longer after wetting events. At the same time, the warming treatment could have accelerated the desiccation of the samples during the early morning dew events through higher evapotranspiration rates. Although it is true that this effect of the warming treatment may happen after any situation involving metabolic activity in the field (e.g., dew, moderate or heavy rain, fog), it could have been of special relevance in the case of dew. This is because dew events are normally quick and short activity events that do not provide water enough to achieve the maximum water content of the samples in the field (Green et al. 2018), being that way more sensitive to higher evapotranspiration. Despite of these characteristics, dew has been shown to be a key water source in dryland biocrusts to obtain adequate C balances in the long term and to avoid extended periods of desiccation (Baldauf et al. 2021; Green et al. 2018; Pintado et al. 2010).

The significant reduction in the length of the active period during the spring observed under the warming treatment is linked to a reduced *Fv*/*Fm*, an indicator of PSII efficiency and of the potential for carrying out photosynthesis (Gauslaa et al. 2012; Vivas et al. 2017). The mean *Fv*/*Fm* observed during the spring in the warming treatment (~0.3) is an indicator of strong physiological stress in green algae (Heber et al. 2010; Lan et al. 2012; Wu et al. 2013) and its reduction with respect to control (Figs. 4d and S3b) could be driven by two mechanisms: (i) reduced photosynthetically active periods during the day (Figs. 4a and 5) and (ii) longer active periods during the nights, which promote extra dark respiration (Lange 2003). Both processes, which lead to C starvation, are triggered in the warming treatment, but our findings (i.e., statistical differences in the length of active periods between treatments in spring during the day and lack of them during the night) point to mechanism (i) as being particularly relevant to explain the *Fv*/*Fm* values observed (Fig. 4d). However, it has been shown that the violaxanthin cycle might be activated also in dark conditions under different type of stressors in brown algae (Fernández-Marín et al. 2011), and this mechanism could be also involved in the reduction of *Fv*/*Fm* found. The increased length of metabolic activity during the nights with warming observed could be related with the slightly higher mean moisture found during the winter (Fig. 2a).

Overall, the models developed show that increasing temperature and decreasing moisture negatively affected the metabolic activity of *Psora*, especially during spring (Figs. S4 and S5). Another remarkable observation is the cessation of the metabolic activity of *Psora* when temperatures reach 0 °C (Figs. S4 and S6). This indicates that *Psora* becomes inactive when the soil surface freezes, but at the same time is able of exploiting melted water from ice early in the morning after sunrise. This suggests an interesting mixed adaptation pattern for crustose lichens in some semi-arid areas that are not exposed very often to extreme cold situations as is the case of this study (see Table S2 for detailed information regarding microclimatic lichen surface temperatures during the winter; see also Kappen 1988; Kappen et al. 1996). Although this mixed pattern mentioned deserves detailed comparative analyses, it seems to be different to previous studies involving cold-adapted lichens, that can show clearly lower temperature values during activity (Schroeter et al. 1994) and optimal physiological status between 0 °C and − 3 °C (Marečková et al. 2019; Raggio et al. 2016; Schroeter et al. 2011).

Several studies have highlighted the deleterious effects of warming over biocrust communities in the field (Darrouzet-Nardi et al. 2015; Guan et al. 2019; Maestre et al. 2013; Rutherford et al. 2017). The time lapses taken to observe significant changes in key community attributes such as cover or species richness vary in relation with the region and biocrust constituent considered, but negative effects are typically observed from three years after experimental set up (Escolar et al. 2012; Ferrenberg et al. 2015; García-Palacios et al. 2018; Ladrón de Guevara et al. 2018; Maestre et al. 2015). These negative effects of warming have to be triggered somehow by physiological responses, that accumulated on time could drift into clear deleterious effects over the communities as those described in the long-term experiments. Maphangwa et al. (2012) found significant decreases in discontinuous measurements of *Fv/Fm* in lichens from drylands exposed to warming. Our results agree with them and go a step forward by providing a continuous temporal resolution and additional relevant performance indicators (e.g., percentage of time being metabolically active on a daily basis). We propose that the observed reduction of this percentage of metabolic activity when environmental conditions (i.e., lichen surface temperature, relative moisture, and PAR) allow net photosynthesis is a key driver of physiological harm induced by simulated warming. Interestingly, Maphangwa et al. (2012) found a higher impact of warming on biocrust-forming lichens in inland (drier) vs. coastal environments. This fact points to water limitation as clear enhancer of the impact of warming over the functional performance of lichens which is strongly aligned with our results. Significantly, habitat-specific features based in the length of the periods with water limitations could be related with the gravity of the impact over biocrusts communities in the long term (i.e., appearance of growth reduction and irreversible cover losses). Future studies combining climatic manipulation, long term monitoring of chlorophyll *a* fluorescence and simultaneous gas exchange measurements could help to quantify real C losses, providing an answer to the questions still open regarding this relevant issue.

## Conclusions

The use of manipulative experiments, like that used here, coupled to the continuous monitoring of physiological performance and microclimatic conditions can provide key mechanistic insights to understand the impacts of climate change on biocrust-forming lichens. In addition, detailed temporal analyses (i.e., diurnal versus nocturnal and seasonal versus annual periods differentiation) are of particular importance to understand responses of biocrust constituents to environmental changes. Our findings indicate that warming and associated reductions in air relative moisture immediately above the thallus monitored will cause a reduction in the metabolic activity when environmental conditions allow net photosynthesis of the biocrust-forming lichen *Psora decipiens*. They also provide a physiological mechanism that can explain the observed responses of biocrust-forming lichens to warming in the field, because the reduction mentioned implies a monitored reduction of both Yield and *Fv/Fm* when activity conditions are warmer during the spring. Combined with the water limitations typically from drylands, this physiological response will impair the C balance of this species by drastically reducing the potential capacity of PSII under climate change, negatively affecting the essential ecosystem functions it provides. As biocrusts are essential regulators of atmosphere-soil interactions in drylands, any damage affecting their growth and cover will have unavoidable harmful consequences over soils stability and state of health.

## Supplementary Information

ESM 1 (DOCX 2866 kb)

## Data Availability

The datasets that support the findings of this study are openly available via Figshare (Raggio et al. 2021) – Doi: 10.6084/m9.figshare.13663616.

## Abbreviations

ΦPSII: Effective quantum use efficiency of PSII in the light
AEMET: Agencia Estatal de Meteorología (Spanish Meteorological Agency)
a.s.l.: Above sea level
C: Carbon
CCOL: Climate Change Outdoor Laboratory of Rey Juan Carlos University
Fm: Maximal fluorescence
Fv: Variable fluorescence
*Fv/Fm*: Effective quantum use efficiency of PSII under dark conditions
GLMMs: Generalized Linear Mixed-Effect Models
LMMs: Linear Mixed-Effect Models
M: Relative moisture
OTC: Open Top Chamber
PAR: Photosynthetically Active Radiation
PSII: Photosystem II
S.L.: Sociedad Limitada (Limited company)
T: Lichen surface temperature
