# Supplementary material for: Continuous monitoring of chlorophyll a fluorescence and microclimatic conditions reveals warming-induced physiological damage in biocrust-forming lichens
Source: Plant Soil. 2022 Sep 9;482(1-2):261–76. doi: 10.1007/s11104-022-05686-w (PMC9870970; doi:10.1007/s11104-022-05686-w)
Supplement: Supplementary file 1 — (DOCX 2866 kb) [file 11104_2022_5686_MOESM1_ESM.docx]

**Supplementary Information**

**Continuous monitoring of chlorophyll *a* fluorescence and microclimatic conditions reveals warming-induced physiological damage in biocrust-forming lichens**

José Raggio^1*^, David S. Pescador^1,2*^, Beatriz Gozalo^3^, Victoria Ochoa^3^, Enrique Valencia^2^, Leopoldo G. Sancho^1^, Fernando T. Maestre^3,4^

* Shared co-first authorship. These authors contributed equally to this work.

^1^Departamento de Farmacología, Farmacognosia y Botánica, Facultad de Farmacia, Universidad Complutense de Madrid, Madrid, Spain.

^2^Departamento de Biología y Geología, Física y Química Inorgánica, Universidad Rey Juan Carlos, Móstoles, Madrid, Spain.

^3^Instituto Multidisciplinar para el Estudio del Medio “Ramon Margalef”, Universidad de Alicante, Carretera de San Vicente del Raspeig s/n, 03690 San Vicente del Raspeig, Spain

^4^Departamento de Ecología, Universidad de Alicante, Carretera de San Vicente del Raspeig s/n, 03690 San Vicente del Raspeig, Spain

David S. Pescador (🖂)

e-mail: david.sanchez@urjc.es

**Appendix I** Microclimatic differences between control and warming conditions. Characterization of OTC microclimatic conditions

Microclimatic conditions experienced by biocrust-forming lichens are key to understand their distribution and adaptation to environmental conditions, especially in habitats with harsh climatic conditions such as drylands ([Green et al. 2007](#_ENREF_1)). In our study, it can be seen how open top chambers (OTCs) increased temperature during all seasons and during diurnal and nocturnal periods, but with statistically significant differences only during the diurnal periods (Fig. 2a). Although the temperature rise in our study is quantitatively higher during the spring in the warming *versus* the control conditions, this difference is significant also in winter and autumn when temperatures are lower, indicating that the impact of temperature rising is not only focused on the warmer periods of the year. At the same time, if the temperature rise provided by the OTC is considered as a percentage of the maximum value, the OTCs increase more the temperature during the winter than during the spring (Table S2). It can be seen also how the rise of temperature during the nocturnal periods provided by the OTC is low, ranging between 0.29-0.64 °C along the three seasons analysed (Table S2). In relation with mean relative moisture, the values are higher during the nocturnal period in the warming treatment, albeit significant differences were found only during the winter, something that very likely contributes to the higher activity values observed during the nights (Fig. 2c, 2d and 4d). The explanation to this pattern could be linked to artificial humidity retention on the chamber surface during the coldest situations. During the diurnal periods there is an opposite effect, with OTC decreasing humidity in all seasons. This effect is a logical consequence of the warming treatment, which is related with a reduction of the length of the periods with high moisture ([Ladrón de Guevara et al. 2014](#_ENREF_2); [Maestre et al. 2015](#_ENREF_3)) and is expected to happen as a consequence of climate change. Indeed, large decreases in relative humidity over mainland Spain, greatest in spring and summer, have been reported from 1961 to 2011 ([Vicente-Serrano et al. 2014](#_ENREF_5)). The analyses of PAR registered over *Psora decipiens* thalli shows how the OTC samples receive a lower amount of PAR during the day (around the 8% of the incident radiation; Table S2 and Fig. S2), but this difference is not statistically significant during any season. The radiation filtering caused by OTCs has a lower impact in high radiation habitats such as the study area because light is not a limitation for the physiological performance of biocrust-forming lichens, and the excess of light is normally not used by cryptogamic communities that are inactive under these situations ([Raggio et al. 2014](#_ENREF_4)).

**References**

Green TA, Schroeter B, Sancho LG (2007) Plant life in Antarctica. In: F Pugnaire, F Valladares (eds) Functional plant ecology. CRC Press, Boca Raton, USA.

Ladrón de Guevara M, Lázaro R, Quero JL, Ochoa V, Gozalo B, Berdugo M, Uclés O, Escolar C, Maestre FT (2014) Simulated climate change reduced the capacity of lichen-dominated biocrusts to act as carbon sinks in two semi-arid Mediterranean ecosystems. Biodiversity and conservation 23: 1787-1807. https://doi.org/10.1007/s10531-014-0681-y.

Maestre FT, Escolar C, Bardgett RD, Dungait JA, Gozalo B, Ochoa V (2015) Warming reduces the cover and diversity of biocrust-forming mosses and lichens, and increases the physiological stress of soil microbial communities in a semi-arid *Pinus halepensis* plantation. Frontiers in microbiology 6: 865. https://doi.org/10.3389/fmicb.2015.00865.

Raggio J, Pintado A, Vivas M, Sancho L, Büdel B, Colesie C, Weber B, Schroeter B, Lázaro R, Green T (2014) Continuous chlorophyll fluorescence, gas exchange and microclimate monitoring in a natural soil crust habitat in Tabernas badlands, Almería, Spain: progressing towards a model to understand productivity. Biodiversity and conservation 23: 1809-1826. https://doi.org/10.1007/s10531-014-0692-8.

Vicente-Serrano SM, Azorin-Molina C, Sanchez-Lorenzo A, Morán-Tejeda E, Lorenzo-Lacruz J, Revuelto J, López-Moreno JI, Espejo F (2014) Temporal evolution of surface humidity in Spain: recent trends and possible physical mechanisms. Climate dynamics 42: 2655-2674.

**
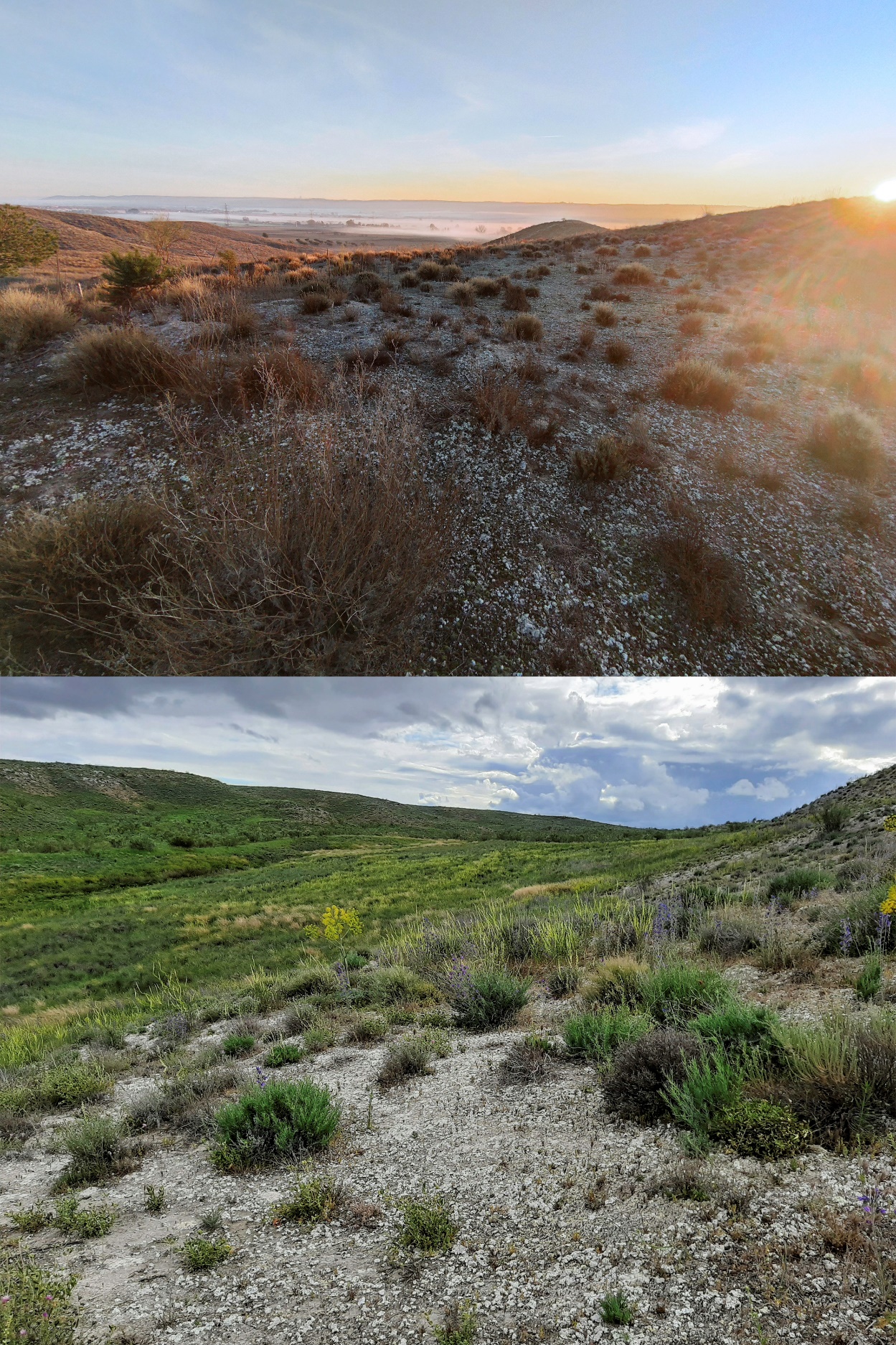
**

**Fig. S1** Pictures of gypsum outcrops where the lichen fragments were collected, located about 50 km south of the CCOL (lat. 40°02′ N, long. 3°32′ W; 590 m asl) and characterized by perennial vegetation patches and biocrusts dominating plant interspaces.

**Table S1.** Summary of lichen richness and species composition of the microcosms monitored in this study. Please note that in all cases the species monitored was *Psora decipiens* (PD). BZ: *Buellia zoharyi*, DD: *Diploschistes diacapsis*, FS: *Fulgensia subbractaceata*, SL: *Squamarina lentigera* and TS: *Toninia sedifolia*.

| ID | Warming treatment levels | Lichen Richness | Species |
| --- | --- | --- | --- |
| 1 | Control | 1 | PD |
| 2 | Control | 6 | BZ, DD, FS, PD, SL, TS |
| 3 | Control | 3 | BZ, PD, SL |
| 4 | Warming | 6 | BZ, DD, FS, PD, SL, TS |
| 5 | Warming | 3 | DD, PD, TS |
| 6 | Warming | 3 | BZ, PD, SL |

**Table S2.** Summary of main quantitative microclimatic differences (mean, standard deviation – sd, treatment differences and percentage of change) observed between control and warming conditions through different temporal periods for temperature, relative moisture and photosynthetically active radiation.

| Lichen surface temperature (°C) | | |  |  |  |  |  |
| --- | --- | --- | --- | --- | --- | --- | --- |
| Day Period | Season | CONTROL | | WARMING | | Treatment differences | Percentage change |
|  |  | Mean | sd | Mean | sd |  |  |
| Diurnal | Autumn | 16.72 | 7.53 | 19.80 | 9.10 | -3.08 | -18.45 |
| Diurnal | Winter | 12.18 | 6.36 | 15.18 | 7.79 | -3.00 | -24.59 |
| Diurnal | Spring | 20.88 | 7.90 | 25.63 | 10.02 | -4.75 | -22.76 |
| Nocturnal | Autumn | 9.29 | 4.37 | 9.65 | 4.46 | -0.36 | -3.92 |
| Nocturnal | Winter | 4.22 | 3.87 | 4.52 | 3.77 | -0.29 | -6.94 |
| Nocturnal | Spring | 10.81 | 4.73 | 11.45 | 4.77 | -0.64 | -5.88 |
| Diurnal | Whole time series | 16.49 | 8.09 | 20.11 | 9.95 | -3.62 | -21.96 |
| Nocturnal | Whole time series | 7.67 | 5.10 | 8.07 | 5.17 | -0.39 | -5.14 |
| Diurnal and Nocturnal | Whole time series | 11.89 | 8.02 | 13.54 | 9.78 | -1.65 | -13.87 |
| Relative moisture (%) | |  |  |  |  |  |  |
| Day Period | Season | CONTROL | | WARMING | | Difference | Percentage change |
|  |  | Mean | sd | Mean | sd |  |  |
| Diurnal | Autumn | 54.68 | 27.50 | 50.38 | 29.72 | 4.30 | 7.87 |
| Diurnal | Winter | 52.90 | 24.28 | 48.45 | 27.42 | 4.45 | 8.42 |
| Diurnal | Spring | 35.48 | 21.53 | 30.15 | 22.77 | 5.33 | 15.02 |
| Nocturnal | Autumn | 81.94 | 18.44 | 84.16 | 20.99 | -2.23 | -2.72 |
| Nocturnal | Winter | 83.45 | 13.64 | 88.55 | 14.32 | -5.10 | -6.11 |
| Nocturnal | Spring | 63.88 | 19.21 | 65.41 | 21.15 | -1.53 | -2.40 |
| Diurnal | Whole time series | 47.96 | 26.06 | 43.21 | 28.33 | 4.75 | 9.90 |
| Nocturnal | Whole time series | 78.56 | 18.65 | 81.72 | 20.74 | -3.16 | -4.02 |
| Diurnal and nocturnal | Whole time series | 63.93 | 27.20 | 64.23 | 31.10 | -0.30 | -0.47 |
| Incident Photosynthetic Photon Flux Density (µmol m-2 s-1) | | | | | | |  |
| Day Period | Season | CONTROL | | WARMING | | Difference | Percentage change |
|  |  | Mean | sd | Mean | sd |  |  |
| Diurnal | Autumn | 458.5 | 392.8 | 420.6 | 353.2 | 37.9 | 8.3 |
| Diurnal | Winter | 480.6 | 421.0 | 429.3 | 348.8 | 51.3 | 10.7 |
| Diurnal | Spring | 834.5 | 637.1 | 771.2 | 569.6 | 63.3 | 7.6 |
| Diurnal | Whole time series | 585.8 | 520.8 | 536.4 | 462.8 | 49.3 | 8.4 |

**
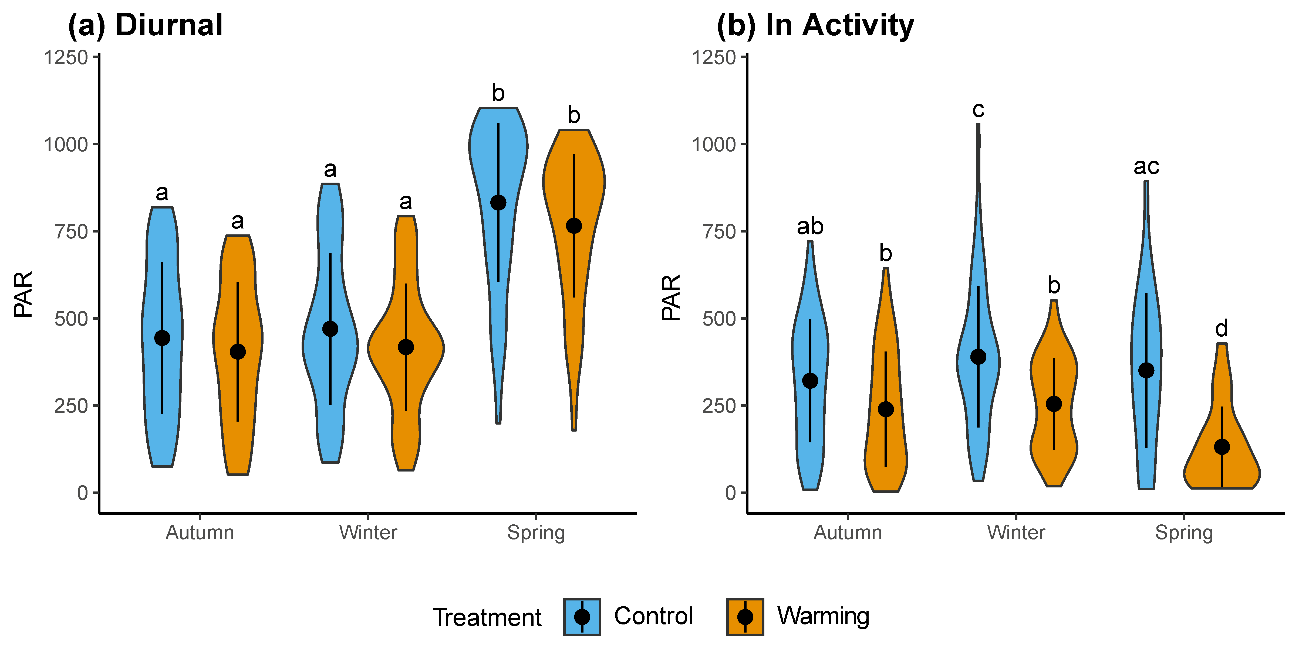
**

**Fig. S2.** Violin plots showing the distribution of daily PAR recorded for autumn, winter and spring seasons for A) the diurnal period (PAR > 0) and B) when the individuals were active (i.e., Yield > 0). Black dots and vertical lines inside each violin represent mean PAR ± standard deviation by warming treatment level and season. Letters above each violin plot indicate significant differences (p < 0.05, Tukey Contrasts post-hoc test) after a Mixed-Effect model. 1482 and 1111 observations were used for the analysis of a and b panels respectively.

**
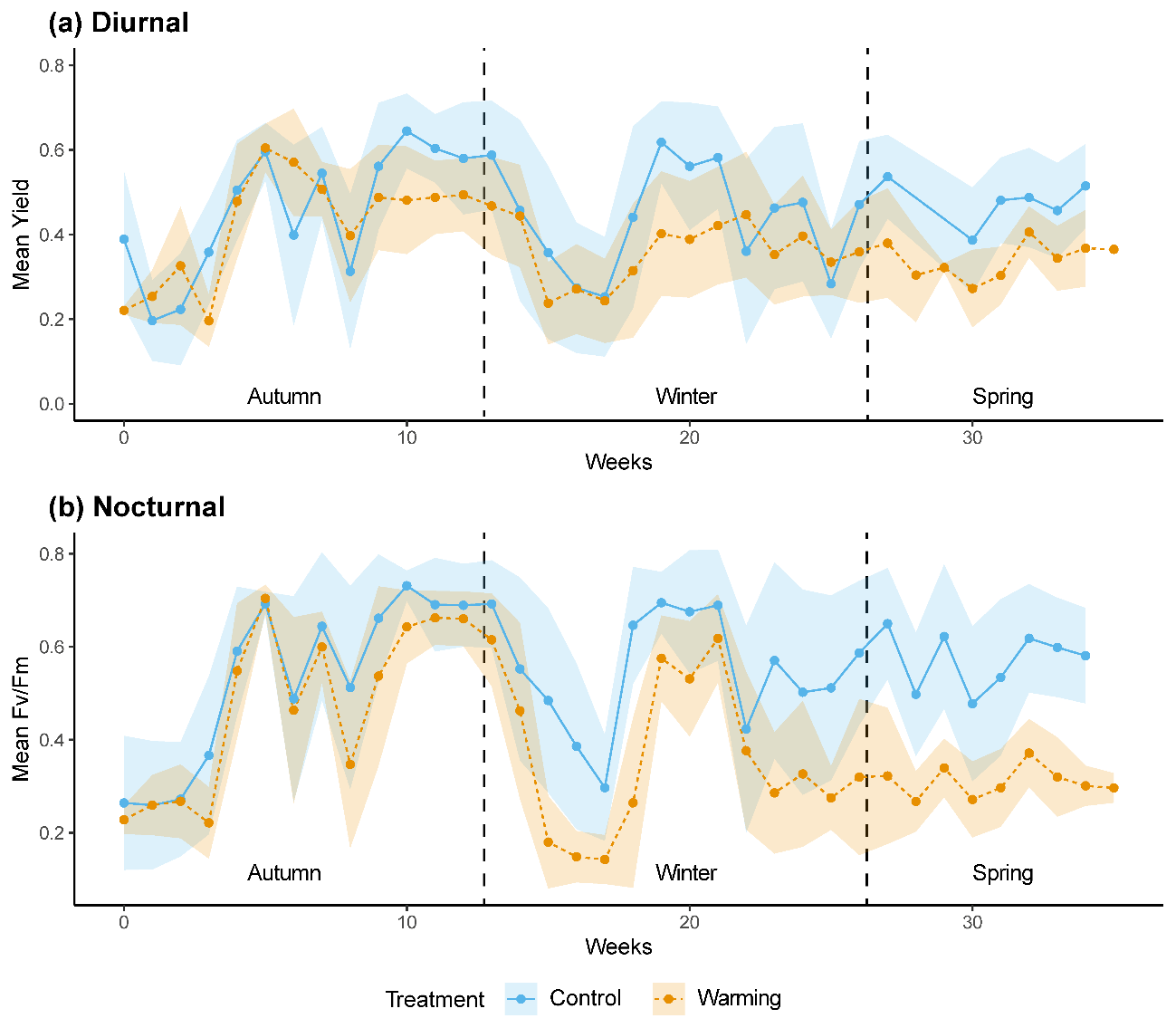
**

**Fig. S3.** Mean weekly Yield (PAR > 0 measurements, upper graph) and *Fv*/*Fm* (PAR = 0 measurements, lower graph) over the course of the experiment. Data are means ± standard error (coloured range) for diurnal and nocturnal periods. The values shown correspond to situations of metabolic activity only.


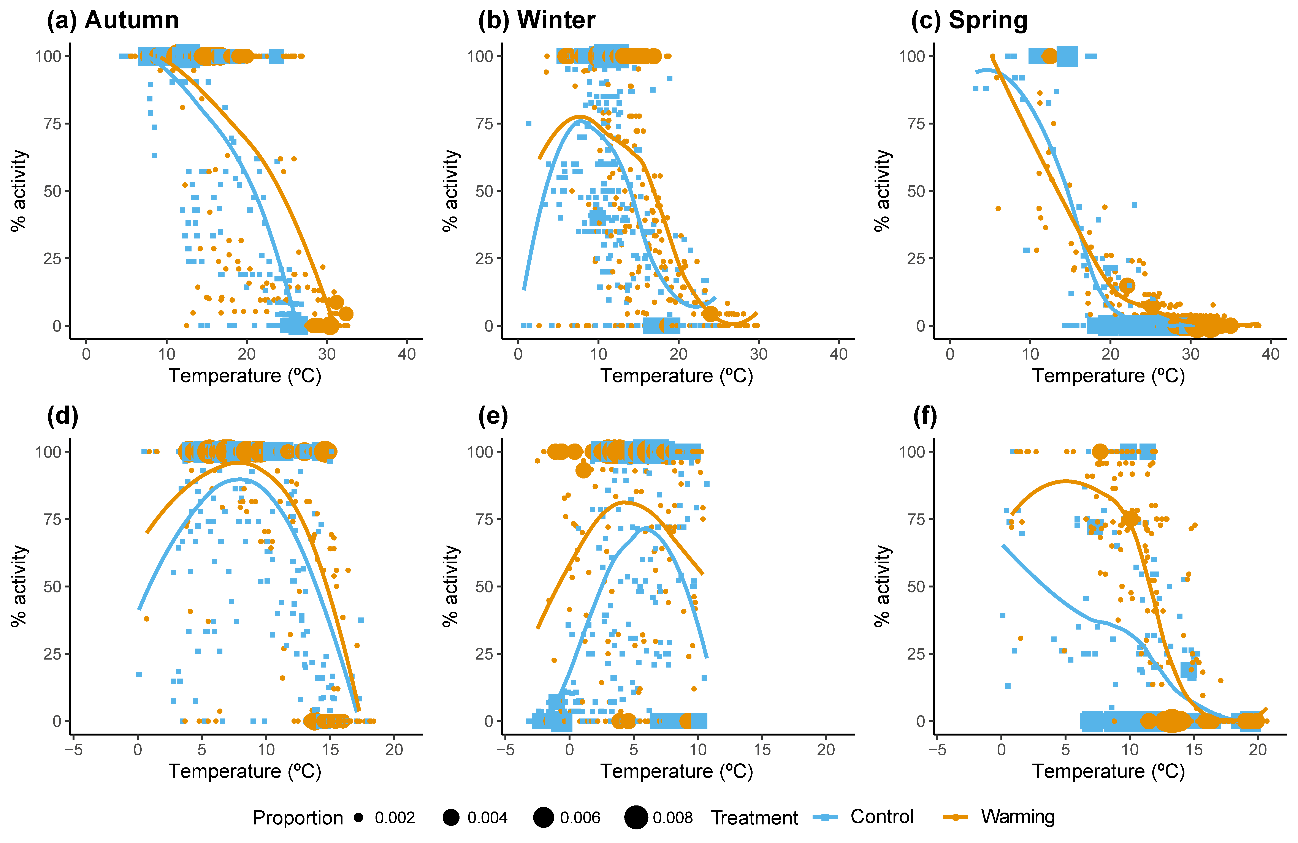


## **Fig. S4.** Relationships between the daily % of activity and temperature. Observations are shown for the diurnal (PAR > 0; upper panels) and nocturnal (PAR = 0; lower panels) periods for autumn (a, b), winter (b, e) and spring (c, f) seasons under control (blue dots) and warming (orange dots) conditions. The size of dots represents the proportion of observations at each temperature. The solid lines represent the smoothed trend fitted by a Local Polynomial Regression Fitting (LOESS) under control (blue lines) and warming (orange lines) conditions. Number of observations were 546, 540 and 396 for autumn (a-d), winter (b-e) and spring (c-f) respectively.


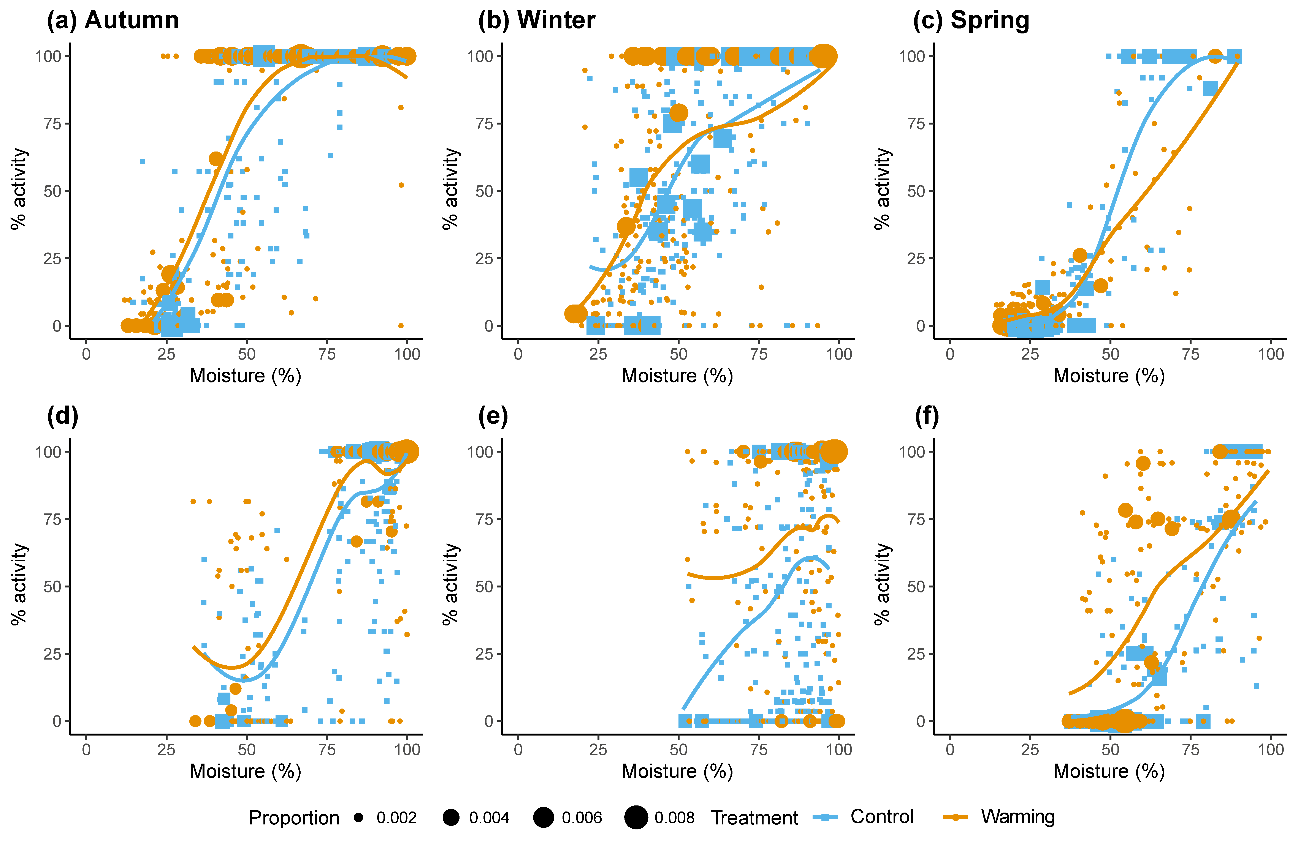


## **Fig. S5.** Relationships between the daily % of activity and air relative moisture. Observations are shown for the diurnal (PAR > 0; upper panels) and nocturnal (PAR = 0; lower panels) periods for autumn (a, b), winter (b, e) and spring (c, f) seasons under control (blue dots) and warming (orange dots) conditions. The size of dots represents the proportion of observations at each relative moisture. The solid lines represent the smoothed trend fitted by a Local Polynomial Regression Fitting (LOESS) under control (blue lines) and warming (orange lines) conditions. Number of observations were 546, 540 and 396 autumn (a-d), winter (b-e) and spring (c-f) respectively.


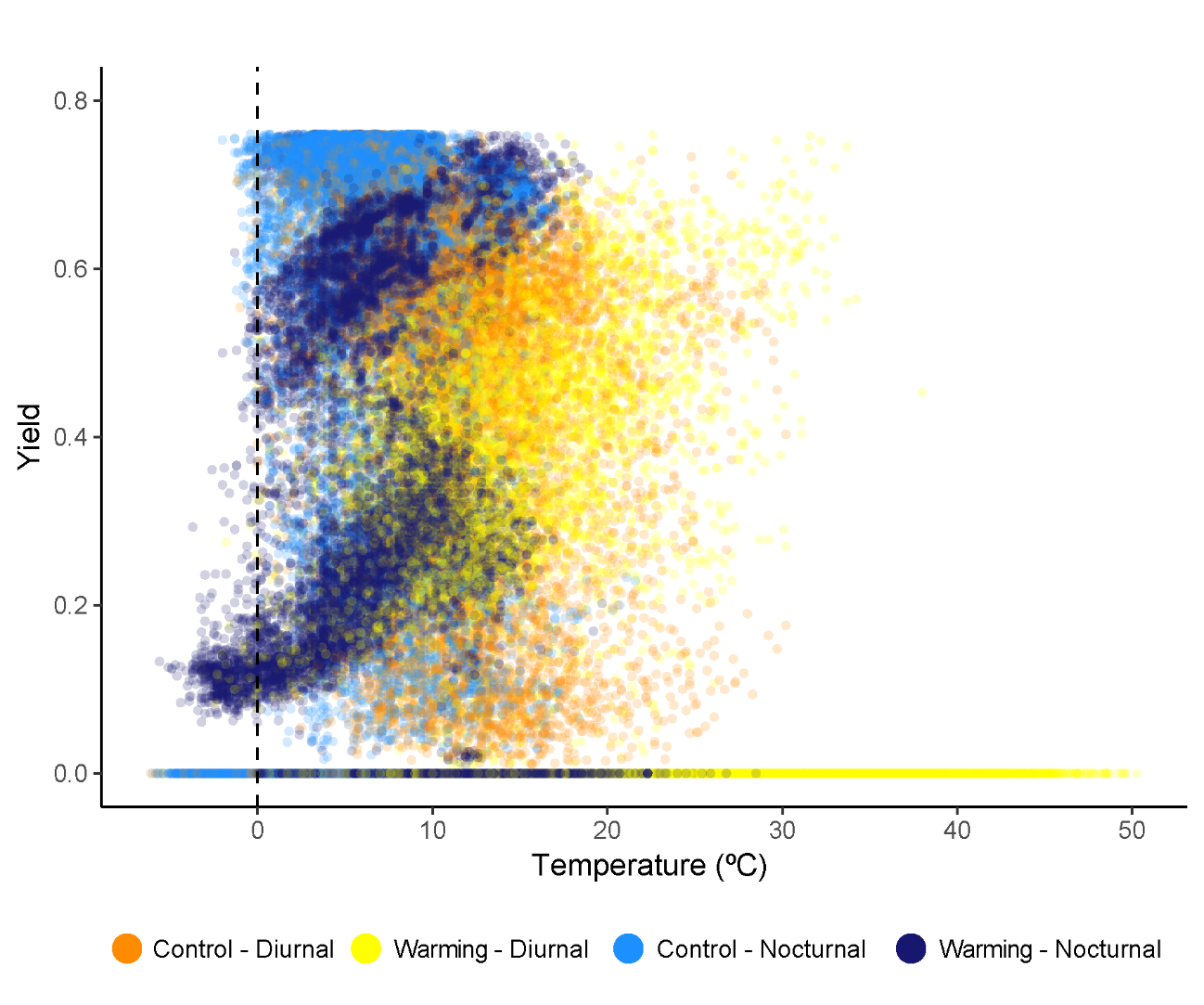


**Fig. S6.** Yield (diurnal periods) and *Fv/Fm* (nocturnal periods) observed across the temperature range recorded during the experiment. Temperature conditions range involving metabolic inactivity are represented in the horizontal bar linked to 0 Yield / *Fv/Fm* values for diurnal and nocturnal periods, respectively.
